# Depression in psychosis: where does it come from?

**DOI:** 10.1192/j.eurpsy.2025.2233

**Published:** 2025-08-26

**Authors:** M. A. Andreo Vidal, M. Calvo Valcárcel, M. P. Pando Fernández, P. M. Gimeno, M. Fernández Lozano, B. Rodríguez Rodríguez, N. Navarro Barriga, M. J. Mateos Sexmero, A. Monllor Lazarraga, L. Rojas Vázquez, G. Lorenzo Chapatte, M. Ríos Vazquero, F. J. González Zapatero, L. del Canto Martínez, C. Rodríguez Valbuena, O. Martin Santiago

**Affiliations:** 1 Psychiatry, Hospital Clínico Universitario de Valladolid, Valladolid, Spain

## Abstract

**Introduction:**

Many patients suffering from schizophrenia have symptoms suggesting depression during the course of their illness. It can appear both in the prodrome of a psychotic decompensation and in the acute phase, as well as after its resolution. But is it part of the disease itself? Is it an experiential reaction to the assumption of the sickness or is it an independent entity? Can it be produced or exacerbated by antipsychotics?

**Objectives:**

This case study aims to analyze the clinical presentation of depressive symptoms in a patient with schizophrenia.

**Methods:**

A review of the literature on affective symptomatology which may occur in psychosis.

**Results:**

A 34-year-old male with a history in Mental Health since the age of 16, with diagnosis of paranoid schizophrenia. He has presented at least 5 depressive episodes and several severe self-harming attempts. He is on treatment with olanzapine, clonazepam, quetiapine and aripiprazole.

During a follow-up, he reports intensification of low mood in the last few weeks due to sentimental break-up, clinophilia and social isolation. He spends the day in his room with the curtains lowered, he has neglected his personal hygiene, and verbalizes thoughts of death. He shows poor functioning, slowed thinking and lack of energy.

His mother reports that he has had self-aggressive behaviors, such as hitting his face and eating his faeces. Sensory and perceptual disturbances are not excluded. Given the current depressive affective state and risk of commiting suicide, it is decided to admit him to the hospital and to start treatment with fluoxetine.

A few weeks after hospital discharge, he continues with poor functioning and isolation, but his mood is better and his thoughts of death have disappeared.

**Conclusions:**

Although clear differentiation between depressive and psychotic symptomatology has been classically described, both symptoms are often associated. Affective symptoms can be part of different stages of the disease, secondary to medication, due to insight phenomena or part of schizoaffective disorder and psychotic depressions.

Depressive symptomatology can also be confused with the presentation of negative symptoms. They both share clinical manifestations such as anergy, social isolation and lack of interest; but while in depression there is a sad mood, in negative symptons there is emotional flattering. Also, positive symptomatology can simulate social withdrawal, usually seen in depression.

Depression in an acute phase has historically been related to a better prognosis, although several studies indicate that depression in a chronic phase causes a higher risk of suicide and relapses. Therefore, early diagnosis and treatment are essential.

In our case, the patient suffers from major affective symptoms regarding his life situation, which may be overlapped by isolation due to a likely positive symptomatology, without dismissing possible negative symptomatology as a result of many years of evolution of his disease.

**Disclosure of Interest:**

None Declared

